# Non-pharmacological, psychosocial MAKS-s intervention for people with severe dementia in nursing homes: results of a cluster-randomised trial

**DOI:** 10.1186/s12877-022-03695-z

**Published:** 2022-12-28

**Authors:** André Kratzer, Kristina Diehl, Olaf Gefeller, Sebastian Meyer, Elmar Graessel

**Affiliations:** 1 Centre for Health Services Research in Medicine, Department of Psychiatry and Psychotherapy, Uniklinikum Erlangen, Friedrich-Alexander-Universität Erlangen-Nürnberg (FAU), Schwabachanlage 6, 91054 Erlangen, Germany; 2 Institute of Medical Informatics, Biometry, and Epidemiology (IMBE), Friedrich-Alexander-Universität Erlangen-Nürnberg (FAU), Waldstraße 6, 91054 Erlangen, Germany

**Keywords:** Severe dementia, Psychosocial intervention, Behavioural and psychological symptoms of dementia, Quality of life, Nursing home, RCT

## Abstract

**Background:**

Severe dementia is one of the most challenging conditions when caring for people in nursing homes. A manualised non-pharmacological, psychosocial group intervention especially adapted to the needs of people with severe dementia (PWSDs) is currently still lacking. To close this gap, we adapted the evidence-based multicomponent non-pharmacological MAKS intervention (**M**otor stimulation, **A**DL stimulation, Cognitive [german: **K**ognitive] stimulation, and **S**ocial functioning in a group setting) to the special needs of PWSDs called the **MAKS-s** intervention, where the **s** stands for **s**evere dementia.

**Methods:**

In a prospective, multicentre, cluster-randomised trial with a waitlist control group design, 26 nursing homes comprising 152 PWSDs were randomly assigned to either the MAKS-s intervention group (IG) or control group (CG) – 121 PWSDs were still alive after the 6-month intervention period (t6) and included in the intention-to-treat (ITT) sample. The two primary outcomes, behavioural and psychological symptoms (BPSDs, measured with NPI-NH) and quality of life (QoL, measured with QUALIDEM), and the secondary outcome, activities of daily living (ADLs, measured with ADCS-ADL-sev), were assessed at baseline (t0) and at t6. Mixed ANOVAs were computed to investigate possible effects of the MAKS-s intervention on the outcomes.

**Results:**

In the ITT sample, BPSDs and QoL did not change significantly over time, and group assignment did not affect them, although the IG participants had significantly better overall QoL than the CG participants. ADLs decreased significantly over time, but group assignment did not affect them. Analyses in the per protocol (PP) sample showed comparable results, with the exception that the IG participants showed a significantly greater increase in BPSDs than the CG participants did.

**Discussion:**

Under the situational conditions of the Covid-19 pandemic, no beneficial effects of the MAKS-s intervention on BPSDs, QoL, or ADLs were observed. This finding also means that under ‘normal circumstances’ (i.e., if there had been no pandemic), we could not make any statements about the effect or non-effect of MAKS-s. In order to be able to address the hypotheses formulated here, the study will have to be repeated incorporating helpful experiences of the present study.

**Trial registration:**

10.1186/ISRCTN15722923 (Registered prospectively, 07. August 2019).

## Background

Severe dementia is one of the most challenging conditions when caring for people in nursing homes, as more than one third of nursing home residents are affected by severe dementia [1–3]. Severe dementia is associated with such great cognitive decline that even basic activities of daily living are compromised [4]. Most people with dementia (PWDs) live in nursing homes until they die [5], which means that even people with very severe dementia in the terminal phase of the disease with a score of 0 on the Mini-Mental State Examination (MMSE), complete dependence in activities of daily living (ADLs), complete incontinence and immobility, and severe impairments in communication skills [6] need to be cared for and meaningfully engaged. However, a lack of meaningful activity for people with severe dementia (PWSDs) is often reported by professional caregivers in nursing homes, as it is difficult to integrate PWSDs into existing group activities [7]. Despite the finding that multicomponent interventions for PWDs are more effective than single interventions [8], international guidelines for treating dementia and especially behavioural and psychological symptoms of dementia (BPSDs) describe only individual interventions for the subgroup of PWSDs, whereas multicomponent non-pharmacological group interventions are missing from the list [9–11]. Accordingly, a recent meta-analysis found only a few high-quality randomised controlled trials (RCTs) that evaluated non-pharmacological group interventions for people with moderate to severe dementia, with moderate to low evidence of beneficial effects on ADLs and depression [12]. However, a non-pharmacological group intervention specifically adapted to the needs of PWSDs with concrete intervention goals and a structured manual for a standardised implementation is currently still lacking.

To close this existing research gap, we decided to further develop the evidence-based multicomponent non-pharmacological MAKS intervention (Motor stimulation, ADL stimulation, Cognitive [german: Kognitive] stimulation, and Social functioning in a group setting), which has already been scientifically approved in two RCTs for its effectiveness on cognition, ADLs, and BPSDs in people with mild to moderate dementia [13–15]. Therefore the MAKS-s intervention (where s stands for severe dementia) was adapted to the special needs of PWSDs, following the ‘unmet needs model’ by Cohen-Mansfield and colleagues [16]. The ‘unmet needs model’ assumes that, due to dementia-related impairments in cognition and communication, PWDs become less and less able to communicate/satisfy their own needs [16, 17]. Thus, these unmet needs are expressed and ‘communicated’ through various behaviours, such as verbal and physical aggression, aberrant motor behaviour, disinhibition, as well as apathy, depression, and anxiety. These behaviours are also known as BPSDs, which affect over 80% of all people with dementia in nursing homes [18] and nearly all people with dementia over the course of their illness [17]. BPSDs are a major challenge for care and nursing staff, as approximately 27% of caregivers report feeling burdened by these symptoms [19] and spending up to 40% of their working time dealing with such symptoms [20]. However, reducing BPSDs is also an important goal in terms of promoting quality of life (QoL), one of the primary goals in the care of PWDs [21]. A frequently discussed question in this regard is: What factors influence the QoL of PWSDs? O’Rourke and colleagues [22] identified four factors: relationships (together vs. alone), agency in life today (purposeful vs. aimless), wellness perspective (well vs. ill), and sense of place (located vs. unsettled) [22]. Consequently, these factors should be addressed in a non-pharmacological intervention for PWSDs. Beyond this, Cohen-Mansfield identified social interaction, moving, meaningful activities, and sensory stimulation as the most important unmet needs in PWSDs contributing to BPSDs [16]. This is exactly where the psychosocial MAKS-s intervention comes in: The group setting and multimodality enable social interaction and participation, feelings of success, meaningful activities, as well as physical activity.

Therefore, the primary hypothesis of the MAKS-s study was that participating in the 6-month MAKS-s intervention would reduce BPSDs and consequently improve QoL in participants in the intervention group (IG) compared with participants in the control group (CG). Furthermore, a secondary hypothesis was that participating in the 6-month MAKS-s intervention would have beneficial effects on ADLs in participants in the IG compared with participants in the CG. Thus, the current work is presenting possible effects of the MAKS-s intervention on outcomes regarding the primary target group of the MAKS-s study, i.e. PWSD.

## Methods

### Study design

The MAKS-s study was a prospective, longitudinal, multicentre, two-armed cluster-randomised controlled trial with a waitlist control group design. The trial was conducted in 26 nursing homes (clusters) located in five federal states of Germany (Bavaria, Baden-Württemberg, Saarland, Thuringia, and Rhineland-Palatine) between July 2019 and September 2021. The intervention period lasted 6 months (June 2020 – December 2020). Data were collected at baseline (t0) and directly after the 6-month intervention period (t6). All procedures were approved by the Ethics Committee of the Friedrich-Alexander-Universität Erlangen-Nürnberg (Ref. 295_19B). Participation was voluntary, and participants as well as clusters were free to leave the study at any time without repercussions. Written informed consent was obtained from all legal representatives of the participants. The study was registered prospectively on 07 August 2019 at ISRCTN registry (Trial identification number: ISRCTN15722923). For more information about the study design, please see our study protocol by Diehl et al. [23].

### Recruitment

The nursing homes (clusters) were recruited between July 2019 and October 2019. Nursing homes with at least 40 residents were identified by their websites in June 2019, called by phone, and informational material was sent by post. All nursing homes that were interested in participating signed a cooperation agreement, which specified the tasks the nursing homes would fulfill during the study and the financial compensation they would receive for doing so. Recruitment ended after the cut-off of at least 24 participating nursing homes was exceeded. This is because the a priori computed sample size estimation showed that at least 144 PWSDs with 6 participants in each nursing home (i.e. at least 24 nursing homes) should be recruited in order to analyse at least 114 PWSDs in the final intention-to-treat sample after an estimated dropout rate of 20% over a 6-month period. Sample size estimation was based on a ranomised-controlled pilot study with two assessments (t0 and t6) in a single nursing home [24] with an alpha error of 5%, a statistical power of 80%, and an effect size of Cohen’s *d* = 0.52 for the outcome BPSDs (for further details regarding the sample size estimation, please see the study protocol [23]). In each participating nursing home, on-site study coordinators were trained in the study protocol, the instruments, and the procedures they needed to follow to conduct the screening and to act as a contact person between the study headquarters and the nursing home.

### Eligibility of participants

All residents of the participating nursing homes were screened between October 2019 and December 2019. Inclusion criteria were a psychometric verification of severe dementia syndrome (i.e. Mini-Mental State Examination [MMSE] score between 0 and 9) and informed consent. Exclusion criteria were: 1. Mild to moderate dementia (i.e. MMSE score > 9); 2. Cognitive decline due to diseases other than dementia (e.g. schizophrenia or Korsakoff); 3. Severe hearing impairment; 4. Severe visual impairment; 5. Permanently bedridden; 6. History of severe major depression; 7. History of more than one stroke; and 8. No verbal communication in German language possible. Each nursing home was supposed to recruit six participants because the MAKS-s intervention was developed as a group intervention for six PWSDs. If more than six eligible individuals were found in the screening, a random procedure to select six study participants out of all eligible individuals was implemented. To this end, the nursing homes were asked to number the screening forms consecutively. Thereafter, the external Institute of Medical Informatics, Biometrics, and Epidemiology (IMBE) of the Friedrich-Alexander-Universität Erlangen-Nürnberg (FAU) generated a list of random integer numbers using the random number generator RAND and the CEIL function in the SAS software, version 9.4 (SAS Institute Inc., Cary, NC). Using these random number lists, the study headquarters informed the participating nursing homes about the order in which they needed to obtain informed consent from the PWSDs or, if applicable, from their legal guardians. This procedure was carried out in each nursing home until a maximum of six participants were recruited for the study. Due to the onset of the Covid-19 pandemic in Germany in March 2020, the 6-month intervention period that had originally been planned to start in March 2020 had to be postponed until the end of June 2020. During the postponement, some participants who had already been enrolled dropped out. In this case, the nursing homes were asked by the study headquarters to enrol the next participant on the randomised list, i.e. informed consent was obtained after randomisation. If there were no more eligible PWSDs on the nursing home’s randomisation list, no further screening process was conducted.

### Randomisation and blinding

After screening and obtaining informed consent from the legal guardians of all participants, the nursing homes (clusters) were randomly assigned to the intervention or control group. Cluster randomisation was chosen in the present study because a non-pharmacological, psychosocial intervention conducted by trained nursing home staff cannot be withheld from any individual from the organisational unit without fear of contamination [25]. Randomisation was concealed and performed externally by the IMBE, and the only information that was shared consisted of the nursing home code, whether the nursing home participated with a secure (i.e. locked) area (yes/no), the federal state in which the nursing home was located, and the total number of residents. The random allocation was stratified by the three factors ‘location’, ‘existence of a secure area’, and ‘total number of residents’. Within the strata, a minimisation procedure [26], i.e. weighted randomisation employing unequal weights depending on the degree of imbalance between the IG and CG, was implemented using the SAS software, version 9.4 (SAS Institute Inc., Cary, NC) to achieve a balanced distribution of the sizes of the nursing homes between groups. After randomisation, the IMBE transmitted the final group allocation to the study headquarters, which then informed the participating nursing homes of their allocation to the IG or CG.

Because we investigated a non-pharmacological, psychosocial intervention, neither the participants (PWSDs) nor the therapists conducting the MAKS-s intervention could be blinded, though we could assume that the PWSDs were essentially blind to the conditions of the study due to the severity of their disease. However, all data collectors at the study headquarters conducting the proxy-rated tests with the nursing staff (raters) were blind to the allocation of the groups. Additionally, before each interview, raters were informed that their group allocation was confidential and that they should not disclose information about their allocation. Beyond that, the raters (nursing staff) of the proxy-rated instruments could be considered ‘semi-blinded’ because they knew about their group assignment but were not given any specific information about the intervention and were not involved in conducting the intervention.

### Intervention

#### Contents of the MAKS-s intervention

The MAKS-s intervention is a multicomponent group intervention consisting of four elements. First is the social warm-up, including social contact and rituals, such as singing together and some other recurring elements (approximately 10 minutes), followed by a sensorimotor session, comprising basic movements and exercises with hand toys, such as mini bean-bags or spiky massage balls (approximately 20 minutes). Afterwards, a short sequence of cognitive stimulation is performed, mainly consisting of activating unconscious memories by singing songs, feeling things with great tactile appeal, or completing proverbs, poems, rhymes, and fairy tales (approximately 10 minutes). The final component involves training in basic ADLs, such as buttering bread, washing hands, or screwing a nut on a thread (approximately 20 minutes). For further details, please see the study protocol [23].

#### Implementation of the MAKS-s intervention

In every participating nursing home, four MAKS-s therapists received a 2-day training in conducting the intervention. The IG was trained before the intervention period, whereas the CG was trained subsequent to the intervention period and after the t6 data collection. Both groups received a therapy manual as well as standardised materials and structured weekly plans to carry out the intervention (for details, see the study protocol [23]). During the intervention period, MAKS-s should be administered to the IG three times a week for 1 hour in groups of six PWSDs and two of the four trained therapists. Due to the Covid-19 pandemic and associated contact restrictions, as well as staff absences due to illness, not all nursing homes were able to offer MAKS-s three times per week over the entire 6-month intervention period. However, the frequency of MAKS-s sessions per participant in each nursing home was recorded and used to determine the PP sample. Due to staff changes, three therapists of one nursing home were retrained by the study headquarters in June 2020.

### Measures

#### Primary outcome measures

*Neuropsychiatric Inventory – Nursing Home Version (NPI-NH)* [27, 28]*.* The NPI-NH is a proxy-rated instrument for assessing the frequency (1–4) and severity (1–3) of 12 common BPSDs in nursing home residents by interviewing formal caregivers about the behaviours they observed during the past week. The total NPI-NH score ranges from 0 to 144 and is obtained by adding the 12 symptom scores (frequency * severity). Higher scores indicate more pronounced BPSDs. Validity and reliability have been established in several studies [28–30].

*QUALIDEM* [31, 32]. The QUALIDEM is a dementia-specific proxy-rated instrument for assessing QoL by interviewing formal caregivers. In the current study, the 18-item version for PWSDs was used. It contains 18 items covering the following six dimensions: care relationship, positive affect, negative affect, restless or tense behaviour, social relations, and social isolation. All items should be rated on a 7-point scale (0–6, ranging from ‘never’ to ‘very frequently’) by formal caregivers regarding observed behaviour in the past week. According to Dichter et al. [33, 34], the global QUALIDEM score is calculated by adding the single item scores and transforming the sum score into values that range from 0 to 100 (QUALIDEM(%) = QUALIDEM (sum score) * 100 / (6 * n (number of items))). Higher scores indicate better QoL. Validity and reliability have been confirmed in several studies [31, 32, 35, 36].

#### Secondary outcome measures

*Alzheimer’s Disease Cooperative Study Activities of Daily Living Inventory – Severe Impairment Version (ADCS-ADL-sev)* [37]. The ADCS-ADL-sev is a proxy-rated instrument for assessing ADL capabilities in PWSDs by interviewing an informant, e.g. a formal caregiver. The 19 items comprise basic ADLs (e.g. eating and bathing) and complex ADLs (e.g. switching on lights or operating faucets). The items should be rated regarding observed behaviour in the past 4 weeks. The total score ranges from 0 to 54 with higher scores indicating higher capabilities in ADLs. Validity and reliability have been confirmed [37].

#### Other outcome measures

*Mini-Mental State Examination (MMSE)* [38]. The MMSE is the most commonly used screening test for detecting dementia and evaluating cognitive functioning in people with dementia [39]. The total score ranges from 0 to 30, whereas a score below 10 indicates severe dementia according to national and international guidelines [40, 41].

*Sociodemographic data, medical history, and comorbidity index.* The following data on each participant’s medical history was collected by the nursing home staff on the basis of the official nursing documentation at baseline: sociodemographic data (age, sex), care level (theoretical range: 1–5, higher scores indicating a greater need for care), prescribed medication, and diagnoses. Comorbidities were weighted using the updated and validated Charlson Comorbidity Index by Quan et al. [42], with higher scores indicating a higher 1-year mortality risk, whereby a score of 5 is associated with an 85% 1-year mortality risk.

### Data collection

Psychology students (data collectors) were trained at the study headquarters to collect data on PWSDs (NPI-NH, QUALIDEM, ADCS-ADL-sev) in semi-structured interviews with the formal caregivers working in the nursing home. Other variables (e.g. sociodemographic data) were collected from nursing documentation by on-site study coordinators. Data on PWSDs were collected at baseline (t0) and directly after the 6-month intervention period (t6). Data collectors ensured that the proxy raters at t6 were the same as at t0 by designating two proxy raters per nursing home and two alternate proxy raters. Unfortunately, due to illnesses and absence at t6 (i.e. the peak of the second Covid-19 wave in Germany), it was often not possible to interview the same proxy raters as at baseline. To reduce reporting bias, formal caregivers providing data on PWSDs were not involved in conducting the MAKS-s intervention in the nursing home. Otherwise, the data collectors were blinded.

To ensure the quality of the data sources, 5% of the data were subjected to random testing. To demonstrate interrater reliability, the QUALIDEM and the NPI-NH of 8 PWSDs were collected by two independent data collectors and different proxy raters in the nursing homes. With an ICC of .77 for the QUALIDEM sum score and ICCs ranging from .67 to .92 for the subscales, good to very good interrater reliability was obtained according to Koo and Li [43]. No further training of the data collectors in the study’s headquarters was required. The NPI-NH was also collected twice by 8 individuals. The interrater reliability of the sum score of the NPI-NH can be classified as moderate with an ICC of .56 according to Koo and Li [43]. Because of these moderate interrater reliability in the NPI-NH, data collectors were again reminded to explicitly read out the sample questions provided by the NPI-NH for each subscale during the telephone-interview with the proxy-raters in order to provide the same standardized frame of reference for all respondents.

### Statistical analysis

The primary data analysis strategy was intention-to-treat (ITT) according to the CONSORT statement [44, 45], considering all participants who were still alive at the end of the intervention period. As a sensitivity analysis, additional analyses with the per-protocol (PP) sample were computed and compared with the results from the ITT analyses. The three criteria for the PP sample were participation in (1) at least one therapy unit in the week directly before data collection, (2) at least 50% of the therapy units in the last 4 weeks before data collection, and (3) at least 50% of the therapy units in the entire intervention period between t0 and t6. Dropout analyses were calculated to check for differences between participants who dropped out and those who completed the study, using chi-square (χ^2^) tests, Mann-Whitney U tests, and t-tests for independent samples.

Missing scores at t6 (NPI-NH, QUALIDEM, ADCS-ADL-sev) for non-deceased participants were imputed externally by the IMBE using iterative random forest imputation [46]. Compared with the EM algorithm, this non-parametric method avoids questionable assumptions of normal distributions and allows for a larger imputation model with potential interaction effects between predictor variables. Predictive mean matching with *k* = 5 candidates between iterations was used to ensure that imputed values obeyed the observed data range. This imputation scheme was stratified by group and implemented in the statistical software environment R using the ‘missRanger’ package [47]. The imputation model included NPI-NH and QUALIDEM total and subscores, the ADCS-ADL-sev total score, as well as the baseline variables sex, age, care level, and MMSE score. Imputation following the above scheme was applied for six participants in the IG (10% of all IG participants) and one participant in the CG (1.6% of all CG participants) at t6.

The underlying assumptions of parametric tests were checked with the Kolmogorov-Smirnov test (normal distribution) and Levene’s test (homogeneity of variance). While sphericity is always given with only two measurement points and Levene’s tests showed that the assumption of homogeneity of variance could be confirmed, Kolmogorov-Smirnov tests showed that the dependent variables NPI-NH total score, QUALIDEM total score, and ADCS-ADL-sev total score were not normally distributed at either t0 or t6. To improve the fit of the outcome data to a normal distribution, we used a square root transformation for the NPI-NH total score and ADCS-ADL-sev total score and a quadratic transformation for the QUALIDEM total score.

Descriptive statistics (frequencies, means (*M*), and standard deviations (*SD*)) were calculated to describe the clusters (nursing homes) and the baseline characteristics of the study participants (PWSDs). In addition, differences between the two groups (IG vs. CG) were evaluated by computing t-tests, Mann-Whitney U tests, and chi-square (χ^2^) tests to assess the quality of the randomisation.

To test the two primary hypotheses and the secondary hypothesis, mixed ANOVAs with the corresponding dependent outcome variable (NPI-NH total score, QUALIDEM total score, or ADCS-ADL-sev total score), the within-subject variable time (two-fold: t0 and t6), and the between-subject variable group (two-fold: IG and CG) were computed.

A type I error rate (alpha) of less than 5% was considered indicative of statistical significance. However, because we performed two main analyses (i.e. NPI-NH and QUALIDEM) in one sample, we had to adjust for multiple testing. Therefore, we applied the Benjamini-Hochberg method [48], which controls the false discovery rate more efficiently than the simple Bonferroni method. According to the Benjamini-Hochberg method, statistical significance is indicated for the lower *p*-value of two main analyses (ANOVAs) at a type I error rate (alpha) of less than 2.5% and for the higher *p*-value at an alpha of less than 5%. Statistical analyses were computed with the software IBM SPSS Statistics, version 28 (IBM Corp., Armonk, NY).

## Results

### Description of clusters (nursing homes)

The present study’s results pertain to the individual level (i.e. PWSDs). Nevertheless, according to the CONSORT extension to cluster-randomised controlled trials [44], we report the intracluster correlation coefficient (ICC), which can be considered low for the two primary outcomes NPI-NH (ICC = .08), QUALIDEM (ICC = .14), and the secondary outcome ADCS-ADL-sev (ICC = .02). Of the 26 participating nursing homes, 13 were randomly assigned to the IG and 13 to the CG. There were no structural differences between nursing homes in the IG and the CG regarding the mean maximum number of residents living in each nursing home, the number of nursing homes participating with a secure area, and the location in the federal states (see Table 1).

**Table 1:** Baseline characteristics of the nursing homes

Variable	Intervention group (*n* = 13)		Control group (*n* = 13)		Total (*n* = 26)		Test of group differences		
							*F*^a^	*t*^*b*^	*p*
Maximum number of residents*, M (SD)*	109.15	(50.16)	102.46	(52.05)	105.81	(50.19)		0.33	.74
Participation with secure area							0.00		> .99
yes, *n* (%)	3	(5.0)	3	(4.9)	6	(5.0)			
no, n (%)	57	(95.0)	58	(95.1)	115	(95.0)			
Federal state							0.99		> .99
Bavaria, *n* (%)	7	(53.8)	6	(46.2)	13	(50.0)			
Baden-Würt., *n* (%)	2	(15.4)	2	(15.4)	4	(15.4)			
Saarland, *n* (%)	2	(15.4)	2	(15.4)	4	(15.4)			
Thuringia, *n* (%)	1	(7.7)	2	(15.4)	3	(11.5)			
Rhineland-P., *n* (%)	1	(7.7)	1	(7.7)	2	(7.7)			

*Note. Baden-Würt*. Baden-Württemberg, *Rhineland-P.* Rhineland-Palatinate

^a^Fisher-Freeman-Halton test value

^b^t-test (t) value

Otherwise, there were no structural differences between the nursing homes that dropped out between t0 and t6 (*n* = 4) and the remaining nursing homes regarding the mean maximum number of residents living in each nursing home (*t*(24) = −0.60, *p* = .553), the number of nursing homes participating with a secure area (χ^2^(1) = 0.10, *p* > .999), or the location in the federal states (Fisher-Freeman-Halton test, *p* > .999).

### Description of study participants

The 6-month intervention period ran from June 2020 to December 2020. A total of 121 participants were included in the ITT sample and randomly allocated to the IG (*n* = 60) or the CG (*n* = 61).

**Fig. 1:**
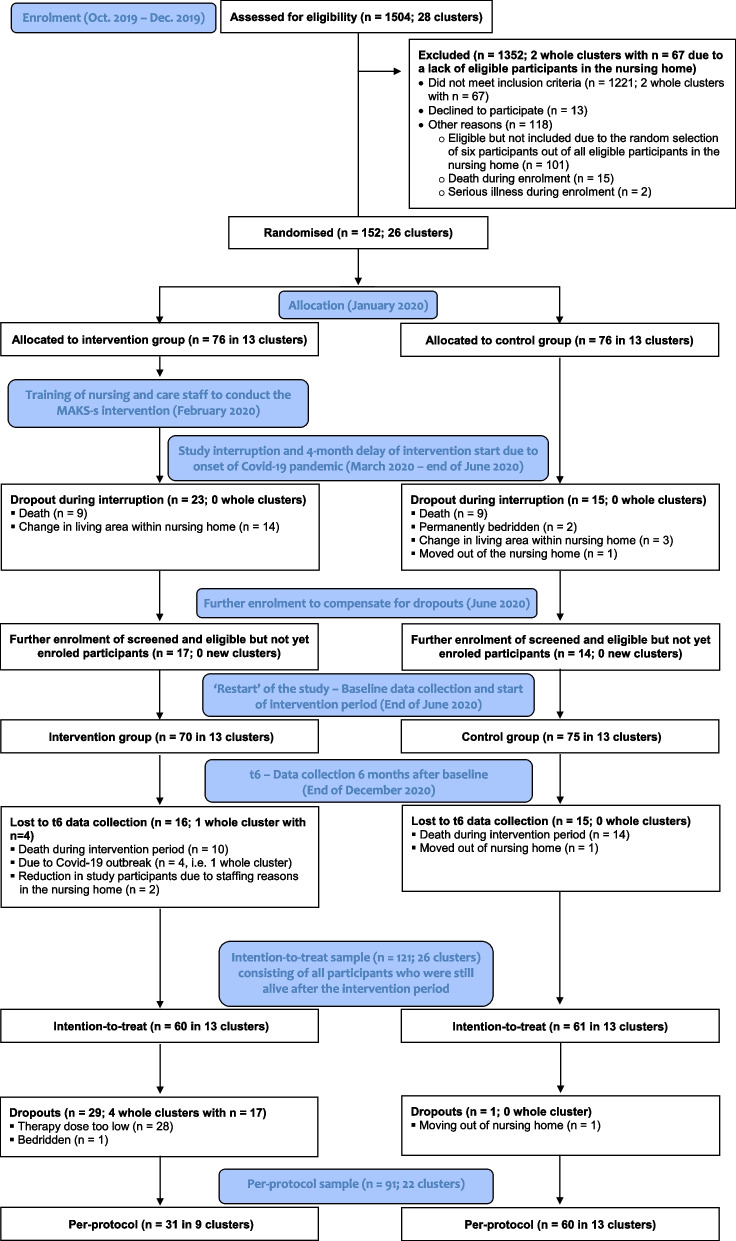
CONSORT Flow Chart of the MAKS-s study. *Note.* Three criteria for a minimum therapy dose were defined a priori: participation in (1) at least one therapy unit in the week directly before data collection, (2) at least 50% of the therapy units in the last 4 weeks before data collection, and (3) at least 50% of the therapy units in the entire intervention period between t0 and t6

The baseline characteristics of the ITT sample are shown in Table 2. There were no significant differences between the groups in the baseline characteristics (see Table 2). Figure 1 shows the study’s CONSORT Flow Chart.

**Table 2:** Baseline characteristics of the study participants (PWSDs)

Variable	Intervention group (*n* = 60)		Control group (*n* = 61)		Total (*n* = 121)		Test of group differences		
							χ^2a^	*t/U*^*b*^	*p*
Age, *M* (*SD*)	85.00	(8.21)	84.25	(5.12)	84.62	(6.81)		0.57	.57
Sex							0.03		> .99
female, *n* (%)	46	(76.6)	46	(75.4)	92	(76.0)			
male, *n* (%)	14	(23.3)	15	(24.6)	29	(24.0)			
Care level, *Mdn* (*IQR*)	4.00	(1.00)	4.00	(1.00)	4.00	(1.00)		1617.50	.22
Antidementia drugs: yes, *n* (%)	14	(23.3)	21	(34.4)	35	(28.9)	1.81		.17
Antipsychotics: yes, *n* (%)	30	(50.0)	34	(55.7)	64	(52.9)	0.40		.53
MMSE sum score, *M* (*SD*)	5.12	(3.30)	4.34	(3.33)	4.73	(3.33)		1.28	.20
Charlson Index *M (SD)*	3.02	(1.38)	3.59	(2.03)	3.31	(1.76)		−1.81	.07

*Note. Mdn* Median, *Care level* higher scores indicate a higher need for care, range: 0–5; *MMSE* Mini-Mental State Examination, lower scores indicate more severe cognitive impairment, and a score between 0 and 9 indicates severe dementia, range: 0–30, *Charlson-Index* Updated and validated Charlson Comorbidity Index by Quan et al., higher scores indicate a higher 1-year comorbidity-related mortality rate, range: 0–24, whereby a score of 5 is associated with an 85% 1-year mortality risk

^a^Chi-square (χ^2^) value from a chi-square test

^b^t-test (t) for interval-scaled variables, Mann-Whitney U test (U) for ordinal-scaled variables

Participants who dropped out between t0 and t6 (*n* = 30) did not differ statistically significant from the remaining PP sample regarding baseline characteristics.

### Primary and secondary hypotheses

The mean values and standard deviations for the two primary outcomes (NPI-NH and QUALIDEM) and the secondary outcome (ADCS-ADL-sev) in the ITT sample at t0 and t6 are presented in Table 3.

**Table 3:** Means, standard deviations, and mixed ANOVA statistics for primary and secondary outcomes in the intention-to-treat sample

Variable	Intervention (*n* = 60)		Control (*n* = 61)		mixed ANOVA^a^				
	*M*	*SD*	*M*	*SD*	Effect	*F ratio*	*df*	*p*	Partial η^2^
NPI-NH									
t0	21.58	19.08	25.56	15.99	G	1.66	1	.200	.01
t6	24.58	16.14	25.34	15.86	T	3.10	1	.081	.03
					G x T	2.72	1	.102	.02
QUALIDEM									
t0	78.64	13.87	71.92	14.75	G	6.08	1	**.015**	.05
t6	75.26	14.62	70.70	15.51	T	3.50	1	.064	.03
					G x T	1.01	1	.317	.01
ADCS-ADL-sev									
t0	12.55	10.30	11.69	8.37	G	0.07	1	.892	.00
t6	10.83	10.58	11.33	9.25	T	9.37	1	**.003**	.07
					G x T	1.01	1	.316	.01

*Note. ANOVA* Analysis of variance, *G* Main effect of group, *T* Main effect of time, *G x T* Interaction effect between group and time, *t0* baseline data collection, *t6* data collection directly after the end of the 6-month intervention period, *Intervention* MAKS-s intervention group, *Control* waitlist control group, *NPI-NH* Total score (frequency x severity) of the Neuropsychiatric Inventory – Nursing Home Version, higher scores indicate more pronounced behavioural and psychological symptoms of dementia, range: 0–144, *QUALIDEM* Total score of the QUALIDEM, higher scores indicate better quality of life, range: 0–100, *ADCS-ADL-sev* Total score of the Alzheimer’s Disease Cooperative Study Activities of Daily Living Inventory – Severe Impairment Version, higher scores indicate higher capabilities in activities of daily living, range: 0–54

^a^ANOVAs were based on scores that were transformed to normality: square root transformation for NPI-NH and ADCS-ADL-sev), quadratic transformation for QUALIDEM

*p-*values < .05 are printed in bold; *n =* 121 (intention-to-treat)

At baseline (t0), ITT sample participants in the IG had a significantly better QUALIDEM score than participants in the CG, (*p* = .009, *d* = 0.49, 95% CI [0.12, 0.85]), whereas they did not differ significantly in terms of NPI-NH, (*p* = .077, *d* = − 0.32, 95% CI [− 0.68, 0.04]), or ADCS-ADL-sev, (*p* = .820, *d* = 0.04, 95% CI [− 0.32, 0.40]).

The mixed ANOVA with the dependent variable NPI-NH did not show a main effect of group, a main effect of time, or an interaction (see Table 3), which means that the two groups had comparable BPSDs, there was no significant change over time in BPSDs, and group assignment did not affect the results.

A mixed ANOVA with the dependent variable QUALIDEM showed a main effect of group that was still significant after the Benjamini-Hochberg correction, but there was no main effect of time or interaction (see Table 3), which means that although IG participants had significantly better overall QoL than CG participants, QoL did not change significantly over time, and group assignment did not affect it.

A mixed ANOVA with the dependent variable ADCS-ADL-sev showed a main effect of time, but there was no main effect of group or interaction (see Table 3), which means that the two groups had comparable ADLs, and although there was a significant change over time in ADLs, group assignment did not affect it.

### Sensitivity analyses in the per-protocol sample

The mean values and standard deviations for the two primary outcomes (NPI-NH and QUALIDEM) and the secondary outcome (ADCS-ADL-sev) in the PP sample at t0 and t6 are presented in Table 4.

**Table 4:** Means, standard deviations, and mixed ANOVA statistics for primary and secondary outcomes in the per-protocol sample

Variable	Intervention (*n* = 31)		Control (*n* = 60)		mixed ANOVA^a^				
	*M*	*SD*	*M*	*SD*	Effect	*F ratio*	*df*	*p*	Partial η^2^
NPI-NH									
t0	18.94	17.58	25.55	16.13	G	1.20	1	.277	.01
t6	27.00	16.20	25.12	15.90	T	10.88	1	**.001**	.11
					G x T	10.90	1	**.001**	.11
QUALIDEM									
t0	80.44	12.25	72.21	14.70	G	5.85	1	**.018**	.06
t6	76.10	13.70	70.90	15.57	T	3.84	1	.053	.04
					G x T	1.37	1	.244	.02
ADCS-ADL-sev									
t0	13.00	9.89	11.70	8.44	G	0.01	1	.937	.00
t6	11.65	11.26	11.26	9.32	T	8.04	1	**.006**	.08
					G x T	0.88	1	.350	.01

*Note. ANOVA* Analysis of variance, *G* Main effect of group, *T* Main effect of time, *G x T* Interaction effect between group and time, *t0* baseline data collection, *t6* data collection directly after the end of the 6-month intervention period, *Intervention* MAKS-s intervention group, *Control* waitlist control group, *NPI-NH* Total score (frequency x severity) of the Neuropsychiatric Inventory – Nursing Home Version, higher scores indicate more pronounced behavioural and psychological symptoms of dementia, range: 0–144, *QUALIDEM* Total score for the QUALIDEM, higher scores indicate better quality of life, range: 0–100, *ADCS-ADL-sev* Total score for the Alzheimer’s Disease Cooperative Study Activities of Daily Living Inventory – Severe Impairment Version, higher scores indicate higher capabilities in activities of daily living, range: 0–54

^a^ ANOVAs were based on scores that were transformed to normality: square root transformation for NPI-NH and ADCS-ADL-sev), quadratic transformation for QUALIDEM

*p*-values < .05 are printed in bold; *n =* 91 (per-protocol)

At baseline (t0), participants in the PP sample in the intervention group had a significantly better QUALIDEM score, (*p* = .004, *d* = 0.61, 95% CI [0.16, 1.05]), and a significantly lower NPI-NH score, (*p* = .024, *d* = − 0.51, 95% CI [− 0.94, − 0.07]), than the control group, but they did not differ in terms of ADCS-ADL-sev, (*p* = .680, *d* = 0.09, 95% CI [− 0.34, 0.53]).

In contrast to the analyses in the ITT sample, a mixed ANOVA with the dependent variable NPI-NH in the PP sample showed no main effect of group, but there was a main effect of time and an interaction that was still significant after the Benjamini-Hochberg correction (see Table 4). This result means that the increase in BPSDs over time was larger in IG participants than in CG participants.

As in the ITT sample, a mixed ANOVA with the dependent variable QUALIDEM in the PP sample showed no interaction effect and no main effect of time, but there was a main effect of group (see Table 4) that was still significant after the Benjamini-Hochberg correction. This result means that although IG participants had a significantly better overall QoL than the CG participants did, QoL did not change significantly over time, and group assignment did not affect it.

Comparable to the analyses in the ITT sample, a mixed ANOVA with the dependent variable ADCS-ADL-sev in the PP sample showed a main effect of time, but there was no interaction effect or main effect of group. This result means that there was a significant decrease in ADLs in the PP sample over time, but group assignment did not affect it.

## Discussion

To our knowledge, the MAKS-s study is the first randomised controlled trial to investigate the effect of a manualised, multicomponent, non-pharmacological, psychosocial group intervention specifically designed for PWSDs on BPSDs, QoL, and ADLs. This is of great importance because a recent meta-analysis found that most studies of non-pharmacological interventions for dementia either did not consider dementia severity or were limited to people with MCI to moderate dementia [12].

It can be stated that under the situational conditions of the Covid-19 pandemic (i.e. social isolation of residents between intervention sessions, reduced group activities such as MAKS-s) and in the light of low adherence of 52%, no beneficial effect of the MAKS-s intervention on BPSDs (measured by NPI-NH), QoL (measured by QUALIDEM), or ADLs (measured by ADCS-ADL-sev) could be observed. However, this also means that no statements can be made about the effect or non-effect of MAKS-s for a ‘normal situation’ (i.e. if there had been no pandemic). Therefore, other possible reasons for the observed null results – besides a possible non-effect of MAKS-s – are discussed below.

Unmet needs in particular are considered to be one of the main causes of BPSDs, and social interaction is considered to be one of the most important unmet needs [16, 17]. Thus, the social isolation of the residents between the intervention sessions caused by Covid-19 restrictions in German nursing homes may have been so severe for the study participants that any positive effect of the MAKS-s intervention may have been overshadowed by this situation. However, it was remarkable that, in contrast to the ITT sample, IG participants in the PP sample showed a significantly lower NPI-NH score at baseline and a significantly greater increase over time in BPSDs than the CG participants did. Nevertheless, The CG showed a high NPI-NH score of approximately 25 at baseline as well as at t6, whereas the IG reached this score at the peak of the second wave of the Covid-19 pandemic in Germany, which unfortunately was at the same time as t6. Since studies investigating BPSDs have shown that NPI-NH total scores usually range from 14 to 25 in different samples of PWDs [28, 30, 49], this observation could be interpreted as a ceiling effect. However, further research is needed to clarify whether this increase was due to the MAKS-s intervention or to the Covid-19 restrictions and the associated social isolation of the residents.

In line with the null results for BPSDs, there was no effect on QoL, a finding that is in agreement with the meta-analysis by Na et al. [12], who also found no effect of non-pharmacological therapies on QoL in PWSDs. These results allow two conclusions: Either the interventions developed so far for PWSDs (including MAKS-s) do not have effects on their QoL, or only a short-term impact on QoL can be achieved in this target group due to the severity of the disease. Further research is needed to clarify this issue.

In contrast to the present results, Na et al.’s recent meta-analysis found positive effects of non-pharmacological interventions on ADLs in PWSDs [12]. However, the study participants in the aforementioned meta-analysis differed considerably in the severity of their disease from the current sample, as they had MMSE scores of 8.8–14.9, whereas the mean MMSE score in the current study was about 4.7, with almost 30% of the participants showing an MMSE score between 0 and 2, which is described in the literature as very severe dementia [50]. Furthermore, the mean ADCS-ADL-sev score of 11–12 in the current sample with more than 50% showing scores below 10 is obviously lower than the mean score (24.5) of the sample in the ADCS-ADL-sev validation study [37]. Therefore, it can be assumed that the proxy-rated ADCS-ADL-sev did not offer sufficient differentiation in the current study’s severely impaired sample. By contrast, the Erlangen Test of Activities of Daily Living (E-ADL) [51], a performance test that we originally planned to use to assess ADLs in this study, is a validated instrument for assessing ADLs in PWSDs and is able to differentiate even in very severely impaired people. Unfortunately, due to the pandemic-related restrictions that had been in place since April 2020 in Germany, this test had to be abandoned, as it requires personal contact.

### Strengths

To our knowledge, this is the first study in Germany to explicitly investigate the effect of a multimodal, non-pharmacological group intervention for PWSDs in nursing homes. The cluster-randomised controlled study design represents a very high quality standard. Due to its natural setting in nursing homes and nursing staff as therapists, the study has high external validity (i.e. validity for the real care situation in nursing homes).

### Limitations

Nevertheless, the present study has some limitations. First, it is not representative of all PWSDs in Germany, as the nursing homes were not randomly selected from the total number of nursing homes. However, due to the diversity of the nursing homes involved (recruitment in five different federal states in urban as well as rural regions, both sheltered and open homes), the data provide a realistic description of the care situation in Germany.

In addition, it cannot be completely ruled out that the on-site study coordinators in the participating nursing homes may not have adhered to the randomisation list when subsequently recruiting study participants after dropouts between March 2020 and June 2020, as the randomisation list only indicated the order in which the eligible persons in the nursing home should be asked for their informed consent one after the other. This may have led to a recruitment bias, i.e. that physically and mentally healthier or fitter people may have been recruited in the nursing homes of the intervention group (as it can be seen in Tables 3 and 4) and that the recruitment efforts in the nursing homes of the control group may have been less motivated due to the group assignment. On the other hand, even a random imbalance cannot be completely ruled out, as this may well occur in such small samples.

Beyond that, the results of the study might be limited by the fact that all outcome variables were collected through proxy-rated assessments that were rated by professional caregivers. As a result, the perspectives of PWSDs could not be included. Taking into account that due to illness and absences, often the same proxy raters could not be interviewed at t6 as at t0, this could have led to biases in the data. In addition, the proxy-raters were not fully blinded to the study conditions, as they knew whether their nursing home residents had been assigned to the intervention or control group.

Furthermore, because the Covid-19 pandemic occurred at the same time as the study, the presence of the pandemic had various effects on how the study could be conducted and thus on the results. Because the pandemic resulted in restrictions on personal contact, only proxy-rated assessment instruments that had been approved by the professional caregivers in the nursing home could be used. Such instruments are less objective than performance tests administered directly with participants or proxy-rated instruments administered by independent clinical raters. In addition, the high physical and psychological stress on professional caregivers caused by the pandemic [52] may have outweighed any positive effects of the intervention, as the post-intervention data were collected at the peak of the second wave of Covid-19 in Germany. In this situation, assessment bias in the sense of a halo effect could be assumed, as the raters were probably burdened by the overall negative situation. Furthermore, due to the isolation of the residents in their rooms in the nursing homes during the intervention period, the raters were hardly able to observe general behavioural patterns, a situation that may have resulted in the low reliability of the data.

Due to these Covid-19 measures, it can be assumed that the social component of the MAKS-s intervention could not unfold sufficiently during the intervention sessions or become permanent in the participants’ everyday lives. However, for a non-pharmacological, psychosocial intervention, it is essential that the behaviours taught and practiced in the therapy sessions, for example, in social interactions as well as in motor activities and ADLs, can also be applied outside the actual therapy sessions (e.g. at lunch together) and thus practiced and consolidated. This fundamental mechanism in the unfolding of the effect of a psychosocial intervention was inhibited in the intervention period by the activity-restricting Covid-19 measures.

And last but not least, only 31 of 60 IG participants received MAKS-s intervention by protocol. This may be due in part to the fact that many participating nursing homes stopped offering MAKS-s, particularly toward the end of the intervention period in November/December 2020 (i.e., at the peak of the second Covid-19 wave in Germany). However, it may also be considered as low adherence on the part of participants, the possible reasons for which would need to be explored in future studies.

## Conclusions

It can be stated that under the situational conditions of the Covid-19 pandemic (i.e. social isolation of residents between intervention sessions, reduced group activities such as MAKS-s) and in the light of low adherence of 52%, no beneficial effects of the MAKS-s intervention on BPSDs, QoL, or ADLs could be observed. However, several issues must be considered when evaluating the results of the present study. The current study’s sample appeared to be significantly more severely impaired than previously studied PWSDs. By far the greatest influence, however, came from the Covid-19 pandemic with all its limitations and restrictions that inhibited the unfolding of potentially positive effects of the non-pharmacological, psychosocial MAKS-s intervention. Beyond that, only 52% of all IG participants received the MAKS-s intervention per protocol. Thus, we cannot make any statements about the effect or non-effect of MAKS-s under ‘normal circumstances’ (i.e. if there had been no pandemic). In order to be able to address the hypotheses formulated here, there is nothing left to do but to repeat the study with the helpful experiences of the present study to optimise the study design: i.e. (1) using performance tests wherever possible instead of proxy-rated instruments, since proxy-rated instruments are of limited use during a pandemic to assess psychological constructs, (2) investigating the short-term impact on QoL in PWSDs, since QoL in general, as measured by QUALIDEM, can be affected by prolonged negative influences such as pandemic restrictions, and last but not least, (3) to ensure that behaviors taught and practiced in therapy sessions can also be applied outside the actual therapy sessions (e.g. at lunch together) and thus practiced and consolidated.

## Data Availability

The data sets used or analysed during the current study are available from the corresponding author upon reasonable request.

## Abbreviations

ADLs: Activities of daily living
ADCS-ADL-sev: Alzheimer’s Disease Cooperative Study Activities of Daily Living Inventory – Severe Impairment Version
BPSDs: behavioural and psychological symptoms of dementia
CG: control group
FAU: Friedrich-Alexander-Universität Erlangen-Nürnberg
IG: intervention group
IMBE: Institute of Medical Informatics, Biometry, and Epidemiology of the Friedrich-Alexander-Universität Erlangen-Nürnberg
ITT: intention-to-treat
MMSE: Mini-Mental State Examination
NPI-NH: Neuropsychiatric Inventory - Nursing Home Version
PP: per-protocol
PWDs: people with dementia
PWSDs: people with severe dementia
QoL: Quality of life
RCT: randomised controlled trial
